# Using the ultra violet C radiation (UVC) led to reduce the airborne microbe in the medical center of North Taiwan

**DOI:** 10.1017/ash.2025.85

**Published:** 2025-09-03

**Authors:** FuChieh Chang, ChiaJung Chan, ShanWen Liu, PeiLun Hung, MingHung Ye

**Affiliations:** 1 Infection Control Center, MacKay Memorial Hospital, Taipei city, Taiwan; 2 Department of Nurse, MacKay Memorial Hospital, Taipei city, Taiwan; 3 Engineering Affairs Office, MacKay Memorial Hospital, Taipei city, Taiwan; 4 Medical Affaris & Planning, MacKay Memorial Hospital, Taipei city, Taiwan

## Abstract

**Introduction:** According to the recommendation of the United States Environmental Protection Agency, the bacteria count of airborne microbe must be under 1500 cfu/ m3. And as we know, the environment is the key factor of the airborne microbe. Traditional, we used air-conditioning to let the air circulate, but this method may be useful in other environments but is not suitable in hospitals. So, there were many new technologies to improve the quality of airborne microbe, just as UVC, plasma, and filtration. In this study, we used the UVC LED to examine the quality of airborne microbes in our meeting room of the emergency room. **Material and method:** We used the impaction method to collect the air for 10 minutes then gathered 1000L air to impact the Tryptone Soy Agar. After collection, we incubated at 37oC for 48 hours the check the bacteria count. So, we used this method to test the quality of airborne microbe before and after adding the UVC-LED (NKFG, Taiwan) to our air conditioner vent in the meeting room of the emergency room. **Result:** Before adding the UVC-LED, the average bacteria count in difference time was from 361 to 443, and after adding the UVC-LED, the average bacteria count in difference time was from 214 to 300, and the percentage of reducing count was from 24% to 40%. **Conclusion:** Due to this study, we though the UVC-LED could improve the quality of the airborne microbe. Otherwise, this technology would not use too much space because of the limitations of the environment.

